# Nurses’ knowledge, attitudes, and perceived challenges toward artificial intelligence applications in patient care: a descriptive-analytical cross-sectional study

**DOI:** 10.1186/s12912-026-04966-5

**Published:** 2026-07-04

**Authors:** Rehab Ragab Bayoumi Elsayed, Ahmed Mohamed Elmarakby Nagy, Eman Sobhy El-Said Hussein, Reham Adel Ebada Elsayed, Reda Mohamed El-Sayed Ramadan

**Affiliations:** 1 Medical Surgical Nursing Department, Faculty of Nursing, Zagazig University, Zagazig, 44519 Egypt; 2 Medical Surgical Nursing Department, Faculty of Nursing, Ain Shams University, Cairo11566, Egypt; 3 Nursing Department, College of Applied Medical Sciences, Shaqra University, Shaqra, 15518 Saudi Arabia; 4 Medical Surgical Nursing, Alghad Colleges for Applied Medical Science, Dammam, Saudi Arabia; 5 Nursing Department, College of Applied Medical Sciences in Yanbu Governorate, Taibah University, Madinah, 42353 Saudi Arabia

**Keywords:** Artificial Intelligence, Nursing, Knowledge, Attitudes, Perceived challenges

## Abstract

**Background:**

Artificial intelligence (AI) is increasingly being integrated into healthcare systems; however, nurses’ knowledge, attitudes, and perceived challenges play a crucial role in its adoption in patient care. This study aimed to assess nurses’ knowledge, attitudes, and perceived challenges toward AI, examine the relationships among these variables, and explore their associations with demographic characteristics and prior AI training.

**Methods:**

A descriptive analytical cross-sectional study was conducted among 107 nurses working in intensive care, medical, and surgical units at Zagazig University Hospital. A purposive sampling technique was used. Data were collected over two months using structured instruments during morning shifts.

**Results:**

Most participants were aged 25–34 years (55.1%), male (65.4%; reflecting the accessible sample composition), and held bachelor’s degrees (68.2%), with nearly half (49.5%) having 5–10 years of clinical experience. Overall, 68.2% of nurses achieved satisfactory knowledge scores, whereas 88.8% demonstrated positive attitudes toward AI applications. Perceived challenges were mainly related to technical and ethical concerns, particularly the need for continuous system updates, cybersecurity risks, and implementation costs. A statistically significant weak negative correlation was found between nurses’ knowledge and attitudes toward AI (*r* = -0.195, *p* = 0.044). No significant correlations were observed between knowledge and perceived challenges (*r* = -0.162, *p* = 0.095) or between attitudes and perceived challenges (*r* = 0.142, *p* = 0.145). Previous AI-related training was significantly associated with more positive attitudes toward AI (*p* = 0.019), whereas no significant associations were found with knowledge or perceived challenges. Educational level, workplace, and years of experience were not significantly associated with nurses’ knowledge, attitudes, or perceived challenges.

**Conclusion:**

Nurses demonstrated a satisfactory knowledge and generally positive attitudes toward AI applications in patient care, while perceiving moderate implementation challenges. Although previous AI-related training was associated with more positive attitudes, no significant associations were found with knowledge or perceived challenges. The weak negative correlation between knowledge and attitudes suggests that greater awareness of AI may be accompanied by increased concerns regarding its use. Further educational initiatives are needed to enhance nurses’ preparedness for AI integration in clinical practice.

## Introduction

According to WHO’s Global Strategy on Digital Health 2020–2025 report, AI is a branch of computer science that focuses on the emulation of human intelligence processes by machines that function and respond like humans. Globally, the application of artificial intelligence (AI) in healthcare is growing quickly, especially in clinical settings. By 2025, global spending on AI in healthcare is expected to reach over 36 billion USD. Consequently, maintaining digital literacy among nurses is essential to ensure the delivery of safe, efficient, and high-quality patient care and to optimize patient outcomes [1].

As the integration of artificial intelligence in healthcare continues to evolve, its impact on clinical practice is becoming more apparent. Nursing activities, care processes, and workflow management are increasingly shaped by AI-driven technologies. These technologies encompass a range of applications, including virtual health assistants, robotic systems, and predictive analytics based on machine learning [2].

Artificial intelligence offers substantial benefits for healthcare delivery by supporting preventive and reactive care, facilitating early disease detection, enabling individualized treatment planning, and enhancing continuous patient monitoring [3]. AI can also improve diagnostic accuracy, optimize clinical workflows, strengthen disease surveillance, and increase healthcare accessibility [4]. Despite these advantages, successful implementation remains challenging because of concerns related to data quality, system interoperability, professional liability, patient privacy, and ethical considerations [5].

Despite these potential benefits, the integration of artificial intelligence into nursing practice is accompanied by several significant challenges. Key concerns include issues related to data privacy and security, ethical implications of AI use, algorithmic bias, and questions regarding the reliability and transparency of AI-driven decision-making systems. Moreover, organizational and human factors such as resistance to technological change, fear of job displacement, and inadequate preparation or training may further hinder nurses’ acceptance and effective adoption of AI technologies in clinical practice [6].

Given nurses’ direct involvement in patient care, their engagement with artificial intelligence is critical for the successful and sustainable integration of AI into healthcare systems. Nurses’ perceptions constitute a fundamental factor influencing organizational readiness and the effective adoption of technological innovations, with positive perceptions strongly associated with higher acceptance and willingness to incorporate AI into clinical practice [7, 8].

The perceived clinical value of AI in enhancing efficiency, supporting professional decision-making, and optimizing workload further strengthens its potential acceptance. However, persistent concerns regarding professional autonomy, patient safety, accountability in decision-making, and job security may adversely shape nurses’ perceptions, thereby limiting adoption and effective utilization of AI technologies in practice [9].

Although the use of artificial intelligence (AI) in healthcare systems is growing globally, there remains limited evidence on nurses’ knowledge, attitudes, and perceived challenges regarding AI applications in patient care, particularly in developing countries such as Egypt. Previous research in Egypt has primarily explored nurses’ perceptions and attitudes toward AI in specific clinical settings, with less focus on their knowledge levels and the obstacles related to AI implementation in practice [10, 11]. Understanding these dimensions is crucial because nurses are key stakeholders in the successful implementation and integration of artificial intelligence (AI) technologies in healthcare settings. Evaluating nurses’ knowledge, attitudes, and perceived challenges can offer important insights into their preparedness for AI adoption and help identify educational and training needs.

This study contributes to the existing body of knowledge by providing empirical evidence on nurses’ knowledge, attitudes, and perceived challenges regarding AI applications in patient care within the Egyptian healthcare context, where evidence remains limited. Given the rapid integration of AI technologies into healthcare systems, understanding nurses’ preparedness is essential for informing nursing education, designing targeted training programs, and supporting evidence-based strategies for successful AI implementation in clinical practice. Accordingly, this study aimed to assess nurses’ knowledge, attitudes, and perceived challenges related to the use of AI applications in patient care.

## Materials and methods

### Aim of the study

This study aimed to: (1) Assess nurses’ knowledge, attitudes, and perceived challenges toward Artificial Intelligence applications in patient care, (2) Examine the relationship between nurses’ knowledge and their attitudes toward AI applications, and (3) Determine the association between nurses’ knowledge, attitudes, perceived challenges and their demographic characteristics, and (4) Examine whether prior AI training is associated with nurses’ knowledge, attitudes, and perceived challenges.

### Research questions

1. What are nurses’ levels of knowledge, attitudes, and perceived challenges toward Artificial Intelligence applications in patient care?
2. Is there a relationship between nurses’ knowledge and their attitudes toward AI applications?
3. Are nurses’ knowledge, attitudes, and perceived challenges significantly associated with demographic variables?
4. Does prior AI training influence nurses’ knowledge, attitudes, perceived challenges?

#### Study design

A descriptive-analytical cross-sectional design was utilized to assess nurses’ knowledge, attitudes, and perceived challenges toward artificial intelligence (AI) applications in patient care.

#### Setting

The study was conducted in the medical and surgical units, as well as the Internal Medicine, Chest, Neuro, and Cardiac ICUs at Zagazig University Hospital, Zagazig, Al Sharqia Governorate, Egypt.

#### Sampling

A purposive sampling technique was utilized to recruit 107 nurses from the selected clinical units. Nurses working in these units were purposively selected because they were more likely to be exposed to or interact with digital and AI-related applications during routine patient care. Eligible participants were full-time nurses who had worked in the selected units for at least one year and provided informed consent to participate in the study. Formal prior training in AI was not required for participation.

The nursing staff in the selected clinical settings worked under a mandatory rotating shift system that included morning, evening, and night shifts. Therefore, many nurses rotated across different schedules during the study period. Although data collection was conducted primarily during daytime hours for administrative feasibility, the rotating nature of nursing schedules may have allowed participation from nurses with experience across different shifts. Nevertheless, the possibility of selection bias related to shift timing cannot be completely excluded and has been acknowledged as a study limitation.

### Sample size calculation

Because this study was conducted in a specialized healthcare institution with a finite and limited accessible population of eligible nurses (*N* = 150), the minimum required sample size was calculated using Yamane’s (1967) formula for finite populations:

$${\rm{n}}={\rm{N}} / (1+{\rm{N}}({\rm{e}}^{2}))$$

Where:

- *n* = required sample size.
- *N* = total accessible population (150 nurses).
- *e* = margin of error set at 0.05.

Based on this formula, the minimum required sample size was estimated to be approximately 109 participants. A total of 107 nurses participated in the study, representing approximately 98% of the required sample size. Therefore, the final sample was considered adequate to achieve the study objectives.

### Instruments of data collection

Data were collected using four structured questionnaires written in English. The questionnaires were administered individually, and the researchers provided clarification whenever necessary to ensure participants’ understanding of the items and the completeness of responses.

#### Structured interview questionnaire

This tool was developed by the researchers to collect demographic and occupational data of the participants. It consists of two parts: **Part I**: Socio-demographic characteristics, including age, sex, marital status, and educational level. **Part II**: Work-related characteristics, including working unit, years of experience, and previous training related to artificial intelligence (AI).

#### Nurses’ knowledge regarding artificial intelligence

This instrument was developed by the researchers based on a review of relevant literature [12, 13] to assess nurses’ knowledge regarding artificial intelligence (AI). The instrument covered definitions, advantages, disadvantages, healthcare applications, the role of AI in enhancing patient care, and barriers to its implementation in healthcare settings.

The instrument consisted of 28 dichotomous (Yes/No) items designed to assess nurses’ factual and conceptual understanding of AI applications in patient care. Each correct response was assigned one point, while incorrect responses received zero points, yielding a total score ranging from 0 to 28.

A score of ≥ 75% (21 points or higher) was considered indicative of satisfactory knowledge, whereas scores < 75% were classified as unsatisfactory. This cut-off was adopted in accordance with the Modified Bloom’s Taxonomy classification, which is widely used in healthcare and nursing research, where scores ranging from 75% to 100% reflect an adequate level of knowledge. Given the clinical implications of AI utilization in nursing practice, a relatively high threshold was considered appropriate to reflect the level of knowledge required for safe and effective use of AI in patient care.

The reliability of the instrument was established using Cronbach’s alpha coefficient, which demonstrated good internal consistency (α = 0.808).

#### Attitude scale toward artificial intelligence

This scale was developed by the researchers based on a review of relevant literature [14] to assess nurses’ attitudes toward artificial intelligence (AI) technologies. The scale consisted of 12 items designed to evaluate nurses’ acceptance, perceptions, and readiness to use AI in patient care. Responses were measured using a three-point Likert scale (Agree = 3, Neutral = 2, Disagree = 1).

The total attitude score was calculated by summing participants’ responses, yielding a possible score range from 12 to 36. Scores of ≥ 60% were considered indicative of a positive attitude toward AI, whereas scores < 60% were classified as negative attitudes. This cut-off was adopted in accordance with Bloom’s classification and previous nursing studies, where scores above 60% are considered to reflect favorable perceptions and acceptance beyond a neutral position.

The reliability of the scale was established using Cronbach’s alpha coefficient, demonstrating good internal consistency (α = 0.827).

#### Nurses’ perceived challenges toward artificial intelligence

This instrument was developed by the researchers based on a review of relevant literature [15–18] to assess nurses’ perceived challenges regarding the implementation of artificial intelligence (AI) in patient care. The instrument consisted of 15 items designed to evaluate barriers associated with the use of AI in healthcare settings.

Responses were measured using a three-point Likert scale (Agree = 3, Neutral = 2, Disagree = 1). The total score was calculated by summing participants’ responses, with higher scores indicating greater perceived challenges toward AI implementation in nursing practice.

The reliability of the instrument was established using Cronbach’s alpha coefficient, which demonstrated good internal consistency (α = 0.868).

### Tool validity and reliability

The study instruments were reviewed by a panel of seven experts in nursing to establish face and content validity. The panel consisted of four professors in Medical-Surgical Nursing and three assistant professors from the Faculty of Nursing, Ain Shams University. The experts evaluated the instruments in terms of content coverage, clarity, wording, comprehensiveness, format, and overall suitability. Based on their feedback, minor modifications were made, including rephrasing ambiguous statements and deleting two items to improve clarity and content appropriateness.

Reliability analysis was conducted to assess the internal consistency of the study instruments. Cronbach’s alpha coefficients were calculated for all multi-item scales, demonstrating acceptable levels of reliability.

### Pilot study

A pilot study was conducted on 10% of the total sample to estimate the time required for data collection and to assess the feasibility, clarity, validity, and applicability of the study instruments. Based on the findings of the pilot study, the necessary modifications were made. Participants included in the pilot study were excluded from the final study sample, and their data were not included in the final analysis.

### study procedure

Data were collected over a two-month period, from the beginning of October 2025 to the end of November 2025. At the outset of data collection, the purpose of the study and the components of the data collection instruments were clearly explained to the participating nurses. Participants were assured that all collected data would remain confidential and would be used solely for research purposes. Data collection was conducted primarily during morning shifts because this period includes the largest number of nursing staff and encompasses most clinical, educational, and administrative activities within the participating university hospital. In addition, the nursing workforce follows a rotating shift schedule, allowing many nurses to work across different shifts during the study period.

Each interview lasted approximately 25–35 min, and all four instruments were administered in a single session. The collected data were subsequently coded, entered, and analyzed using appropriate statistical methods to assess nurses’ knowledge, attitudes, and perceived challenges regarding the use of artificial intelligence applications in patient care across internal medicine, chest, neurology, and cardiac intensive care units, as well as medical and surgical units.

### Operational definitions

#### Artificial Intelligence (AI) in patient care

Refers to the use of computer-based systems and algorithms capable of performing tasks that typically require human intelligence such as clinical decision support, prediction, data analysis, and automation within patient care settings.

#### Prior AI training

Self-reported participation in any formal or informal training, workshop, course, or educational activity related to artificial intelligence before data collection.

### Statistical analysis

Data were coded, entered, and analyzed using the Statistical Package for the Social Sciences (SPSS), version 24. Descriptive statistics were used to summarize the data. Qualitative variables were presented as frequencies and percentages, whereas quantitative variables were presented as mean ± standard deviation (SD). Prior to inferential analyses, the normality of continuous variables was assessed using the Shapiro–Wilk test. Since the study variables were not normally distributed, nonparametric tests were applied. Spearman’s rank correlation coefficient was used to examine relationships among continuous study variables. Statistical significance was considered at *p* ≤ 0.05.

## Results

The results are organized to describe the characteristics of the study sample and to assess nurses’ knowledge, attitudes, and perceived challenges regarding artificial intelligence (AI), followed by an analysis of the relationships among these variables and their associations with selected socio-demographic and work-related factors.

**Table 1:** Socio-demographic characteristics of the study participants

Items	No	%
Age: < 25 25–34 35–44 45–54	23 59 24 1	21.5 55.1 22.4 1.0
Mean ± SD	30.58 ± 6.17	
Gender: Male Female	70 37	65.4 34.6
Social status: Single Married Divorced	31 75 1	29.0 70.1 0.9
Educational level: Diploma in nursing Technical Institute Bachelor’s degree Master degree PhD	4 17 73 10 3	3.7 16.0 68.2 9.3 2.8

Table 1 shows that the majority of nurses were aged 25–34 years (55.1%), with a mean age of 30.58 ± 6.17 years. Most participants were male (65.4%) and married (70.1%). The majority held a bachelor’s degree (68.2%), while smaller proportions had technical (16.0%) or postgraduate qualifications (master’s: 9.3%, PhD: 2.8%).

**Table 2:** Work-related characteristics of the study participants

Items	No	%
**Work place:** ICU Medical Unit Surgical Unit	54 9 44	50.5 8.4 41.1
**Years of experience:** < 5 From 5–10 From 11–20	33 53 21	30.8 49.5 19.7
**Previous training related to AI** Yes No	21 86	19.6 80.4

Table 2 shows that half of the nurses worked in ICUs (50.5%), followed by surgical units (41.1%), with few in medical units (8.4%). Nearly half had 5–10 years of experience (49.5%), indicating a moderately experienced workforce. Most participants (80.4%) reported no prior AI training, highlighting a notable gap in formal education.

**Table 3:** Total knowledge score of nurses regarding artificial intelligence (AI)

Variable	No	%
**Definition of AI:** Satisfactory Unsatisfactory	31 76	29 71
**Advantage of AI:** Satisfactory Unsatisfactory	96 11	89.7 10.3
**Dis advantage of AI in Nursing:** Satisfactory Unsatisfactory	48 59	44.9 55.1
**Application of AI in Health Care:** Satisfactory Unsatisfactory	76 31	71 29
**Enhancing Patient care:** Satisfactory Unsatisfactory	57 50	53.3 46.7
**Major barriers to the widespread AI in health sector:** Satisfactory Unsatisfactory	61 46	57 43
**Total score:** Satisfactory Unsatisfactory	73 34	68.2 31.8

Table 3 shows variability in nurses’ knowledge across AI domains. High proportions demonstrated satisfactory knowledge of AI advantages (89.7%) and healthcare applications (71%), whereas knowledge of AI definitions was lower (29%). Knowledge of disadvantages was also limited, with 55.1% classified as unsatisfactory. Similarly, knowledge related to enhancing patient care (53.3%) and barriers to AI implementation (57%) showed moderate satisfactory. Overall, 68.2% of the participants achieved the predefined satisfactory knowledge level (≥ 75% of the total knowledge score), indicating acceptable awareness despite gaps in fundamental concepts and limitations of AI.

**Table 4:** Nurses’ attitudes toward artificial intelligence (AI) applications

Variable	Mean ± SD
1- Do you agree that you have good familiarity with artificial intelligence?	2.23 ± 0.68
2- Do you agree that artificial intelligence has useful applications in the healthcare field?	2.57 ± 0.67
3- Do you agree that the diagnostic ability of artificial intelligence is superior to the clinical experience of a human doctor?	1.57 ± 0.75
4- Do you agree that artificial intelligence could replace your job?	1.61 ± 0.77
5- Do you agree that you will always use artificial intelligence when making medical decisions in the future	1.83 ± 0.75
6- Do you agree that artificial intelligence is dangerous	2.11 ± 0.70
7- Do you agree The integration of AI into healthcare will reduce the workload for nurses	2.45 ± 0.69
8- Do you agree that AI has the potential to revolutionize the role of nurses in healthcare delivery	2.55 ± 0.64
9- Do you agree that AI can provide equitable healthcare access, especially in underserved areas	2.37 ± 0.75
10- Do you agree AI minimizes human errors in tasks such as data entry, diagnostics, and financial transactions?	2.52 ± 0.63
11- Do you agree AI will likely lead to significant improvements in the quality of care provided to patients	2.51 ± 0.66
12- Do you agree AI facilitate improvement in nursing profession	2.52 ± 0.63

Table 4 shows that nurses had generally positive attitudes toward AI, with higher mean scores for its usefulness in healthcare (2.57 ± 0.67), potential to transform nursing roles (2.55 ± 0.64), and ability to reduce workload (2.45 ± 0.69). However, low scores for AI surpassing clinical expertise (1.57 ± 0.75) and replacing jobs (1.61 ± 0.77) reflect skepticism toward its replacement role. Moderate concern about risks (2.11 ± 0.70) indicates a balanced perception of AI.

**Table 5:** Total attitude score of nurses toward artificial intelligence (AI) applications

Variable	No	%
Positive attitude Negative attitude	95 12	88.8 11.2
** Mean ± SD**	26.89 ± 4.92	

Table 5 shows that the majority of nurses (88.8%) demonstrated a positive overall attitude toward AI applications, with a mean score of 26.89 ± 4.92. Only a small proportion (11.2%) exhibited a negative attitude.

**Table 6:** Nurses’ perceived challenges regarding artificial intelligence (AI) applications

Variable	Mean ± SD
**Errors, reliability and implementation Challenges**	
I think artificial intelligence systems make many errors	2.23 ± 0.72
Computers can be hacked, which can lead to health care problems	2.57 ± 0.67
Artificial intelligence needs continuous updates.	2.66 ± 0.61
Applications of artificial intelligence cannot be used to provide opinions	2.41 ± 0.72
It is difficult to apply to all patients	2.45 ± 0.69
Data acquisition occurs at stages of artificial intelligence implementation	2.48 ± 0.69
**Human-Machine Interaction and Relationships**	
Artificial intelligence will negatively affect the health care providers-patient relationship	2.31 ± 0.77
It will be difficult to totally replace health care providers’ consultation with digital tools	2.51 ± 0.69
Artificial intelligence reduces the trust and quality of the nursing care providers/patient	2.34 ± 0.79
**Ethical and Social Concerns**	
Organizations use artificial intelligence unethically	2.32 ± 0.78
Artificial intelligence may lead to misuse of critically ill patient data	2.44 ± 0.74
Using artificial intelligence led to elevated costs of the system	2.52 ± 0.69
Cannot identify responsible for causing harm caused by artificial intelligence mistakes	2.48 ± 0.69
Ethical and social issues occur at stages of artificial intelligence implementation	2.40 ± 0.75
**Job Replacement and Trust**	
A longstanding concern regarding artificial intelligence in healthcare is the fear it will replace jobs	2.13 ± 0.83

Table 6 shows that nurses had moderate awareness of challenges related to AI implementation. Higher mean scores were reported for system updates (2.66 ± 0.61), cybersecurity risks (2.57 ± 0.67), and increased costs (2.52 ± 0.69). Concerns were also noted regarding human–machine interaction, particularly the difficulty of replacing healthcare providers, as well as ethical issues such as data misuse and accountability. Job replacement concerns were present but less pronounced (2.13 ± 0.83).

**Table 7:** Correlation between nurses’ knowledge, attitudes, and perceived challenges regarding artificial intelligence (AI)

Variable	Nurses’ Knowledge	Nurses’ Attitude
Nurses’ Attitude	*r* = -0.195*, *p* = 0.044	—
Nurses’ Challenges	*r* = -0.162, *p* = 0.095	*r* = 0.142, *p* = 0.145

*Correlation is significant at the 0.05 level (2-tailed)

Spearman’s rank correlation coefficient was used

Table 7 shows a statistically significant weak negative correlation between nurses’ knowledge and attitudes toward AI applications (*r* = -0.195, *p* = 0.044). However, no statistically significant correlations were found between nurses’ knowledge and perceived challenges (*r* = -0.162, *p* = 0.095) or between attitudes and perceived challenges (*r* = 0.142, *p* = 0.145). The magnitude of this correlation was weak, indicating a small effect size. The non-significant correlations between knowledge and perceived challenges and between attitudes and perceived challenges also reflected weak associations.

**Table 8:** Differences in nurses’ knowledge, attitudes, and perceived challenges according to educational and work-related characteristics (*N* = 107)

Variable	Knowledge (Mean Rank)	Test (*p*)	Attitude (Mean Rank)	Test (*p*)	Challenges (Mean Rank)	Test (*p*)
Educational level		χ² = 6.658 (0.155)		χ² = 0.925 (0.921)		χ² = 2.649 (0.618)
Diploma in Nursing (*n* = 4)	63.75		59.88		54.38	
Technical Institute (*n* = 17)	49.59		57.74		47.09	
Bachelor’s Degree (*n* = 73)	56.79		52.44		56.01	
Master’s Degree (*n* = 10)	37.00		58.60		57.25	
PhD (*n* = 3)	54.83		47.67		33.00	
Workplace		χ² = 5.217 (0.074)		χ² = 1.374 (0.503)		χ² = 1.425 (0.491)
ICU (*n* = 54)	50.87		50.53		57.32	
Medical Unit (*n* = 9)	42.94		57.72		54.44	
Surgical Unit (*n* = 44)	60.10		57.50		49.83	
Years of Experience		χ² = 0.929 (0.629)		χ² = 0.735 (0.693)		χ² = 2.677 (0.262)
< 5 years (*n* = 33)	56.45		55.71		52.06	
5–10 years (*n* = 53)	54.16		51.50		51.29	
11–20 years (*n* = 21)	49.74		57.62		63.88	
Previous AI Training		U = 760.000 (0.164)		U = 604.500 (0.019*)		U = 849.000 (0.671)
Yes (*n* = 21)	47.19		68.21		56.57	
No (*n* = 86)	55.66		50.53		53.37	

*Significant at p < 0.05

Kruskal–Wallis test was used for educational level, workplace, and years of experience. Mann–Whitney U test was used for previous AI-related training

*Significant at *p* < 0.05.

Kruskal–Wallis test was used for educational level, workplace, and years of experience. Mann–Whitney U test was used for previous AI-related training.

Table 8 demonstrates that educational level, workplace, and years of clinical experience were not significantly associated with nurses’ knowledge, attitudes, or perceived challenges regarding AI applications in patient care (*p* > 0.05). Similarly, no statistically significant differences were found in nurses’ knowledge or perceived challenges according to previous AI-related training (*p* > 0.05). However, nurses who had received previous AI-related training demonstrated significantly more positive attitudes toward AI applications than those who had not received such training (U = 604.500, *p* = 0.019).

This finding suggests that previous exposure to AI training may positively influence nurses’ attitudes toward AI, although it does not necessarily improve their knowledge levels or alter their perceptions of implementation challenges.

## Discussion

The successful integration of artificial intelligence into patient care depends not only on technological advancement but also on healthcare professionals’ readiness to adopt and use these systems effectively. Nurses, as primary providers of direct patient care, are expected to interact with AI-supported technologies across various clinical settings. Consequently, examining nurses’ knowledge, attitudes, and perceived challenges toward AI is important for understanding their preparedness for technology-enabled healthcare environments. The current findings are discussed in light of previous research and contemporary challenges surrounding AI implementation in nursing practice.

Discussion of the present study will cover four main areas: First, Socio-demographic and work-related characteristics; Second, Nurses’ levels of knowledge, attitudes, and perceived challenges toward artificial intelligence applications in patient care; Third, Nurses’ knowledge, attitudes and significantly associated with demographic variables; Fourth, Prior AI training in relation to knowledge, attitude, and perceived challenges.

### First: Socio-demographic and work-related characteristics

Findings of this study revealed that more than half of participants were 25–34 years old, predominantly within the early stages of their professional careers. This demographic profile is consistent with [19] who revealed that most participants were 20–40 years old. This suggests that the nursing workforce in many settings is increasingly composed of younger professionals who are generally more exposed to digital technologies.

In terms of gender distribution, the predominance of male participants contrasts with global nursing demographics, where females typically represent the majority. This current finding does not align with [20] who revealed that, highest responses from females. This may be explained by the fact that most of the participants who knew about AI and agreed to participate in the study were males.

Regarding educational level, more than two thirds of participants held a bachelor’s degree. This finding aligns with [19] despite differences in healthcare systems and cultural contexts. The similarity may reflect the global trend toward recruitment of younger nurses who are more familiar with digital technologies and technology-enhanced healthcare environments.

Work-related characteristics further contextualize the findings. A large proportion of nurses were working in the intensive care unit (ICU) and surgical units, environments characterized by high patient acuity and an increasing reliance on technology. This may partially explain the relatively high awareness of AI applications observed in the study. However, a critical finding is that the majority of participants had no prior training related to AI. This finding is consistent with [20] who reported that more than two-thirds of participants had not attended national or international conferences on AI adoption in healthcare. The similarity between the findings may indicate that limited formal exposure to AI education and training remains a common challenge across healthcare settings, despite differences in organizational and geographical contexts.

Moreover, the distribution of years of experience showed that approximately half of the participants were within the 5–10-year range. This finding is partially consistent with [21] who reported that more than half of participants had more than five years of experience.

This distribution may reflect the typical workforce structure in healthcare settings, where a substantial proportion of nurses are in the mid-career stage (5–10 years of experience). This stage is often associated with increased professional stability and retention compared to early-career nurses, who may exhibit higher turnover rates. Additionally, the inclusion of specialized units such as intensive care may have contributed to this pattern, as such settings often require nurses with a moderate level of clinical experience.

### Second: Nurses’ levels of knowledge, attitudes, and perceived challenges toward artificial intelligence applications in patient care

Although participants showed a satisfactory level of AI application and advantage, substantial deficiencies in basic AI concepts, particularly regarding AI definitions and AI disadvantages remained in their understanding of fundamentals of AI . This pattern suggests greater familiarity with the practical use of AI than with its theoretical foundations. This finding is broadly consistent with [21] who reported a moderate level of AI knowledge among participants. From a management perspective, this pattern may be attributed to the functional nature of knowledge in clinical settings, where nurses are more exposed to the practical benefits and applications of AI rather than its underlying concepts or technical foundations. This results in selective knowledge acquisition, where applied aspects are better understood than analytical or theoretical components.

A notable finding in this study is that the majority of participants demonstrated positive attitudes toward AI. This is consistent with previous research, which reported that attitudes toward AI had the highest mean score among participants [22]. This pattern may be attributed to the influence of expectations and prevailing narratives surrounding AI, which often emphasize its potential benefits in healthcare. This may also reflect an optimism bias toward emerging technologies, where individuals express favorable attitudes despite limited hands-on experience or critical understanding.

The current study found that nurses demonstrated moderate awareness of challenges associated with AI implementation. Participants expressed the highest concerns regarding system updates, cybersecurity risks, and increased costs, reflecting the predominance of technical challenges. Concerns related to human–machine interaction, such as the perceived difficulty of replacing healthcare providers and potential impacts on patient relationships, were also noted, alongside ethical considerations including data misuse and accountability for AI-related errors. Job replacement fears were less pronounced, suggesting that workforce displacement was not the primary concern. These findings are consistent with previous research, which reported that technical challenges were identified by less than two thirds of participants, ethical concerns (particularly regarding privacy) by more than half, and fears of job displacement by less than half of participants [23].

This may be explained by the fact that nurses place greater emphasis on ethical and privacy-related challenges due to their direct responsibility for patient safety and the management of sensitive health data. As a result, these concerns are more immediate and tangible than potential risks related to job displacement. Together with the focus on technical and operational issues, these factors help explain why nurses’ perceptions of AI challenges in clinical practice are primarily centered on technical and ethical considerations rather than fears of workforce replacement.

The study revealed a statistically significant weak negative correlation between nurses’ knowledge and attitudes toward AI, indicating that nurses with higher levels of AI knowledge tended to report slightly less favorable attitudes toward AI applications. This finding may suggest that greater knowledge of AI is accompanied by increased awareness of its limitations, ethical concerns, implementation challenges, and potential implications for professional practice. Previous studies have highlighted that increased awareness of AI-related ethical issues, including data privacy, transparency, accountability, and the impact of AI on professional roles, may influence healthcare professionals’ perceptions and acceptance of AI technologies [19]. However, given the weak magnitude of the correlation and the cross-sectional nature of the study, no causal relationship can be inferred.

### Third: Nurses’ knowledge, attitudes and significantly associated with demographic variables

The lack of significant relationships between knowledge or attitudes and demographic variables in the present study contrasts with findings from previous research. For example, a recent study reported significant associations between nurses’ demographic characteristics such as age, gender, years of experience, and educational level and their attitudes toward artificial intelligence [24].

However, this discrepancy may be explained by differences in sample composition and study context. In the current study, the relative homogeneity of the sample, particularly in terms of age and educational level, may have limited variability and reduced the ability to detect statistically significant associations. Furthermore, it is possible that non-demographic factors, such as prior exposure to AI and training, played a more influential role in shaping participants’ perceptions. Therefore, these findings should be interpreted as context-specific rather than contradictory to existing literature.

### Fourth: Prior AI training in relation to knowledge, attitudes, and perceived challenges

Previous AI-related training was significantly associated with more positive attitudes toward AI, while no significant differences were observed in knowledge or perceived challenges. This suggests that prior exposure to AI may improve acceptance and readiness to engage with AI technologies, but training as currently experienced by participants may not be sufficiently comprehensive to improve factual knowledge or reduce implementation concerns. The small effect size indicates that the practical influence of prior training was modest. Therefore, AI education for nurses should combine conceptual knowledge, hands-on exposure, ethical guidance, and clinical application. Previous studies have emphasized the importance of ongoing education, professional development, and tailored training programs in enhancing healthcare professionals’ preparedness and readiness for AI adoption [25]. Similarly, [26] reported that educational interventions had a highly statistically significant positive effect on nurses’ knowledge and attitudes regarding artificial intelligence, highlighting the potential role of structured educational programs in promoting AI acceptance and utilization in nursing practice.

### Limitations

This study has several limitations. Its cross-sectional design prevents establishing causal relationships between knowledge, attitudes, and perceived challenges. The reliance on self-reported measures may introduce response bias. The relatively homogeneous sample could limit the generalizability of the findings, and the absence of qualitative data restricts a deeper understanding of the contextual and experiential factors influencing nurses’ perceptions of AI.

Although nurses in the participating units worked under a rotating shift system, data collection was conducted primarily during morning shifts. Therefore, nurses working predominantly evening or night shifts may have been underrepresented, which could limit the generalizability of the findings.

## Conclusion

In conclusion, the study demonstrated that nurses generally possessed a satisfactory knowledge and positive attitudes toward AI applications in patient care, while perceiving moderate implementation challenges. A statistically significant weak negative correlation was identified between knowledge and attitudes, suggesting that greater knowledge of AI may be accompanied by increased awareness of its limitations, ethical concerns, and implementation challenges. No significant associations were observed between knowledge and perceived challenges or between attitudes and perceived challenges. Previous AI-related training was significantly associated with more positive attitudes toward AI but showed no significant association with knowledge or perceived challenges, indicating that education alone may not be sufficient to address the broader barriers to AI implementation in clinical practice. Furthermore, educational level, workplace, and years of experience demonstrated limited influence on nurses’ knowledge, attitudes, and perceived challenges, suggesting that contextual and organizational factors may play a more substantial role in shaping nurses’ perceptions of AI.

### Recommendations

- **Strengthen AI Education and Training:** Develop comprehensive educational and training programs that enhance nurses’ understanding of AI concepts and provide practical experience with AI applications in clinical settings.
- **Address Organizational and System-Level Barriers:** Healthcare organizations should establish supportive policies, infrastructure, and leadership strategies to facilitate the safe and effective integration of AI into nursing practice.
- **Promote Interdisciplinary Collaboration:** Encourage collaboration among nurses, healthcare administrators, information technology specialists, and policymakers to ensure that AI technologies are aligned with clinical needs and nursing workflows.
- **Enhance Ethical and Regulatory Preparedness:** Educational and institutional initiatives should address ethical concerns related to AI, including data privacy, accountability, transparency, and patient safety.
- **Conduct Further Research:** Future longitudinal and multicenter studies are recommended to explore the influence of organizational culture, technological readiness, and previous exposure to AI on nurses’ perceptions and successful AI implementation.

### Implications for nursing practice and critical care

- Enhanced Readiness: Structured AI training can increase nurses’ confidence and willingness to integrate AI into patient care, particularly in high-acuity areas such as ICUs and surgical units.
- System-Level Interventions: Critical care settings must address infrastructural, regulatory, and ethical challenges to optimize AI adoption and patient safety outcomes.
- Evidence-Based Policy: Insights from this study can guide hospital administrators and policymakers in developing comprehensive strategies that combine education, technological resources, and governance frameworks to support effective AI implementation.
- Professional Development: Fostering AI literacy among nurses can strengthen interdisciplinary collaboration, enhance clinical decision-making, and improve patient care quality in digitally evolving healthcare environments.

## Data Availability

The datasets used and analysed during the current study are available from the corresponding author on reasonable request.

## Abbreviations

AI: Artificial intelligence
ICU: Intensive care unit
SPSS: Statistical Package for the Social Sciences
